# Retraction: Applications of bioconvection for tiny particles due to two concentric cylinders when role of Lorentz force is significant

**DOI:** 10.1371/journal.pone.0323142

**Published:** 2025-05-07

**Authors:** The PLOS One Editors

## Introduction:

The *PLOS One* Editors retract this article [1] due to concerns about potential manipulation of the publication process. These concerns call into question the validity and provenance of the reported results. We regret that the issues were not identified prior to the article’s publication.

VP, MIjK, and NAS did not agree with the retraction. LZ, EREZ, NM, JDC, SUK, and MImK either did not respond directly or could not be reached.
